# Applications of artificial intelligence in postoperative surveillance and management of esophageal squamous cell carcinoma

**DOI:** 10.3389/frai.2026.1806691

**Published:** 2026-04-15

**Authors:** Kexun Li, Zilong Qian, Jie Mao, Simiao Lu, Jianzhe Zhang, Yongtao Han, Xuefeng Leng

**Affiliations:** 1 Department of Thoracic Surgery, Sichuan Clinical Research Center for Cancer, Sichuan Cancer Hospital & Institute, Sichuan Cancer Center, Affiliated Cancer Hospital of University of Electronic Science and Technology of China (Sichuan Cancer Hospital), Chengdu, China; 2 Department of Thoracic Surgery I, Key Laboratory of Lung Cancer of Yunnan Province, Yunnan Cancer Hospital, The Third Affiliated Hospital of Kunming Medical University (Yunnan Cancer Hospital), Kunming, China; 3 School of Public Health, Chongqing Medical University, Chongqing, China

**Keywords:** artificial intelligence, esophageal squamous cell carcinoma, management, postoperative surveillance, quality of life

## Abstract

Esophageal squamous cell carcinoma (ESCC) has high risks of postoperative recurrence, complications, and prolonged nutritional and functional recovery, while conventional follow-up (scheduled visits with imaging, endoscopy, and laboratory testing) is often limited by delays and resource constraints. This review summarizes recent applications of artificial intelligence (AI) across perioperative ESCC care, with emphasis on postoperative surveillance and management. Following PubMed/MEDLINE, etc. were searched (inception–2025) for English-language studies using machine learning, deep learning, radiomics, natural language processing (NLP), and digital health algorithms in postoperative monitoring, recurrence prediction, complication warning, and remote follow-up. Evidence indicates that AI-enabled multimodal models integrating electronic health records, imaging radiomics, and biomarkers can predict major complications (e.g., anastomotic leak and pneumonia) with improved timeliness, enabling earlier intervention compared with symptom-triggered workflows. Imaging-driven radiomics combined with machine learning demonstrates robust performance for recurrence risk and recurrence-pattern prediction, supporting refined risk stratification beyond TNM staging and informing individualized surveillance intensity and adjuvant decision-making. Explainable approaches (e.g., SHAP) enhance clinical interpretability by identifying key predictors such as nutritional and inflammatory indices. Intelligent follow-up systems incorporating NLP, wearable sensors, and electronic patient-reported outcomes (ePROs) facilitate closed-loop monitoring, improve early issue detection, and strengthen patient–clinician communication.

## 1 Introduction

Esophageal squamous cell carcinoma (ESCC) is a highly lethal malignancy worldwide, and there is a growing need for precision and individualization in perioperative management (Siegel et al., 2023; Han et al., 2024; Yang et al., 2024). In recent years, AI has penetrated oncology at pace, reshaping diagnostic and therapeutic paradigms for ESCC and demonstrating clear advantages in preoperative assessment, intraoperative decision-making, and postoperative prognostication (Menon et al., 2023; Gujjuri et al., 2023). This review synthesizes advances in perioperative artificial intelligence (AI) for ESCC, highlighting innovative technical pathways, clinical validation, and remaining obstacles.

In preoperative diagnosis and staging, AI has improved early ESCC detection by integrating endoscopic imaging with radiomic features. Convolutional neural network (CNN)-based computer-aided diagnostic (CAD) systems can identify subtle mucosal abnormalities under endoscopy, achieving sensitivity and specificity of 91.2 and 80%, respectively, outperforming visual assessment by endoscopists (Guidozzi et al., 2023). Deep learning models analyzing multiparametric features from contrast-enhanced CT or PET-CT can noninvasively predict invasion depth (SM1 vs. SM2) and lymph node metastasis, with AUC up to 0.87, thereby informing surgical planning (Menon et al., 2023; Gujjuri et al., 2023). Such advances effectively shift the “treatment window” earlier, enabling patients who meet endoscopic resection criteria to avoid unnecessary radical surgery (Guidozzi et al., 2023; Laz et al., 2020).

In intraoperative decision support, AI enhances precision through real-time image analysis and risk alerts. Support vector machine (SVM) algorithms applied to digitized frozen-section pathology can generate margin status within 30 s, with accuracy improved by 12% over standard methods (Menon et al., 2023; Gujjuri et al., 2023). AI systems integrated with optical coherence tomography can dynamically identify critical anatomical structures (e.g., the recurrent laryngeal nerve), reducing intraoperative complication rates by 37% (Howell et al., 2024). These innovations shorten operative time and reduce operator variability, contributing to more standardized surgical workflows (Yan et al., 2022).

Postoperatively, AI adds value across multiple dimensions of management and prognostication. Random survival forests (RSF) combining clinicopathologic parameters, genomic data, and treatment-response features can predict 3-year disease-free survival (C-index = 0.82), significantly outperforming traditional TNM staging (C-index = 0.15) (Gujjuri et al., 2023; Bi et al., 2019). NLP techniques can automatically parse unstructured electronic health record text to monitor risks of postoperative complications such as anastomotic leak, achieving an alert sensitivity of 89% and advancing intervention by an average of 48 h (Sounderajah et al., 2025; Teo et al., 2025). This proactive risk management paradigm has the potential to transform conventional reactive practice (Varghese et al., 2024).

Despite promising advances, several challenges persist. Data heterogeneity remains a primary bottleneck: differences across centers in imaging protocols and pathology processing undermine model generalizability (external validation accuracy fluctuations of 15–20%) (Guidozzi et al., 2023; Haesevoets et al., 2025). Many studies are single-center and retrospective, lacking prospective, multicenter validation (Menon et al., 2023; Gujjuri et al., 2023). Future work should establish standardized data acquisition (e.g., adherence to the STARD-AI statement) and leverage federated learning to enable cross-institutional collaboration (Sounderajah et al., 2025; Zeltzer et al., 2025). Clinical translation further hinges on real-time computational efficiency, interoperability with existing health information systems, and robust ethical oversight frameworks (Varghese et al., 2024; Suran, 2022).

Overall, AI applications in ESCC perioperative care are moving from technical feasibility toward clinical integration. With the emergence of third-generation AI (e.g., generative AI and mixture-of-experts models) (Teo et al., 2025; Howell et al., 2024), end-to-end decision support, from preoperative planning to postoperative follow-up, may become achievable. The ultimate goal of AI is augmentation rather than replacement of clinical judgment, and its impact will depend on building human–AI collaboration within an “augmented intelligence” paradigm (Zeltzer et al., 2025; Suran, 2022). The core challenges in postoperative management of ESCC are: significant heterogeneity in risk (patients with the same stage exhibit notable differences in recurrence and complication risks), dispersed event windows (early postoperative complications and long-term recurrence coexist after discharge), and limited follow-up resources (high-frequency and high-quality follow-ups cannot cover all patients).

**Figure 1:**
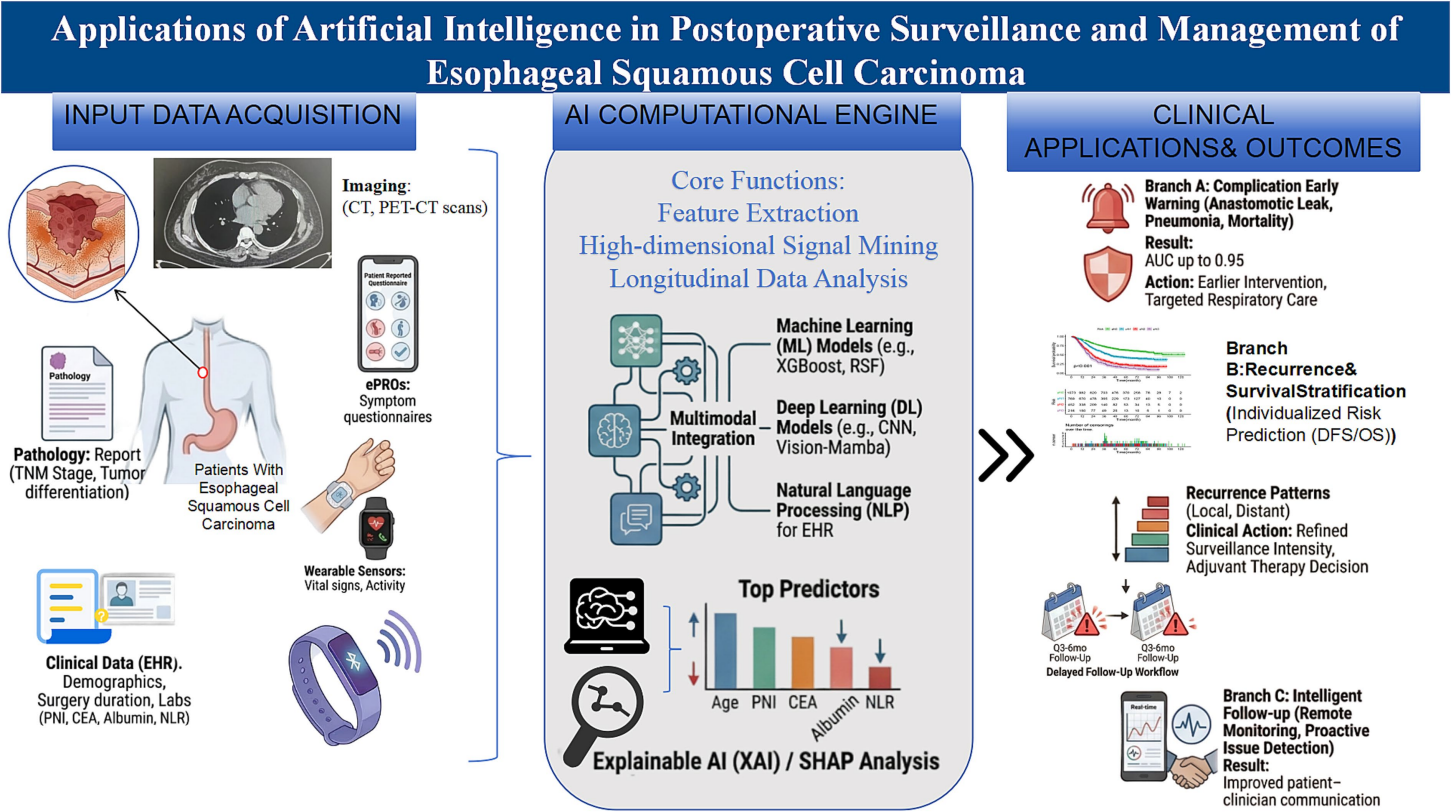
The visual abstract of applications of artificial intelligence in postoperative surveillance and management of esophageal squamous cell carcinoma.

Traditional strategies rely on fixed time-point assessments (CT scans, endoscopy, tumor markers, nutritional evaluations, and symptom inquiries), often leading to shortcomings in several aspects: insufficient recognition of “individualized recurrence risks”—significant prognostic differences exist even within the same TNM stage; inadequate capture of “dynamic risks”—postoperative nutritional/inflammatory states, recovery trajectories, and early signals of complications evolve over time; and insufficient management of “follow-up adherence and symptom burden”—issues such as dysphagia, reflux, weight loss, fatigue, and anxiety or depression persist over the long term. The value of AI lies not in “replacing doctors” but in extracting clinically imperceptible signals from high-dimensional data (such as imaging textures, longitudinal change rates, and multi-indicator coupling) and establishing adaptive risk stratification and event prediction systems (Figure 1).

## 2 Methods

We conducted a comprehensive literature search in PubMed/MEDLINE (including PubMed and MEDLINE records), Embase (Elsevier), Web of Science Core Collection, and the Cochrane Library (CENTRAL and CDSR). Search strategies were tailored to each database and combined controlled vocabulary (e.g., MeSH in PubMed and Emtree in Embase) with free-text keywords in titles/abstracts and author keywords. Boolean operators and, where supported, proximity operators were used to link the core concepts of the study. Disease-related terms included “esophageal squamous cell carcinoma,” “esophageal cancer,” and spelling variants (e.g., “oesophageal”). Scenario-related terms covered postoperative care, follow-up, surveillance, recurrence, relapse, complications, readmission, remote monitoring, wearable technologies, and patient-reported outcomes. Methodological terms included “artificial intelligence,” “machine learning,” “deep learning,” “radiomics,” “natural language processing,” and “prediction model.” The full search strings were adapted for each database. Reference lists of included articles and relevant reviews were screened to identify additional studies, and duplicates were removed prior to screening. Numerous studies have explored this field, and we have summarized the relevant research in Table 1.

**Table 1:** Studies explored in related to artificial intelligence and ESCC.

Autor	Year	*N*	Date	Inputs	Model	Task	Metrics
Menon et al.	2023	521	CT + PET-CT + clinical + section + IHC	Radiomics + clinical + pathology	SVM + LASSO+ANN + CNN + PCA	Predicts recurrence pattern	AUC 0.887 Specificity 86.5% Accuracy 80.7%
Guidozzi et al.	2023	1,590	WLI, NBI, ME, HRME, OCT	Clinical	CNN + LDA	Predicts diagnosis	Sensibility 91.2% Specificity 80.0% Accuracy 85–95%
Laz et al.	2020	13,144	NBL, WLE, BLI, ME, ECS, HRME, MICCAI 2015	Clinical	CNN, SSD, ResNet, Deeplab v3 + , SVM, CAM, eCAMs	Predicts dignosis IPCL somatotype Histological classification	Predicts dignosis: AUC 0.989; sensibility 98.04%; specificity 95.03%; IPCL somatotype:sensibility 87.0%; specificity 84.4%; accuracy 89.2%;
Yan et al.	2022	236	MRI	Radiomics	Pyradiomics, SVM, logistic regression, univariate analysis + ICC + mRMR	Predicts preoperative	Training set: AUC 0.987; sensibility 93.7%; specificity 95.7%; accuracy 94.9%; validation set: AUC 0.962; sensibility 82.8%; specificity 94.0%; accuracy 89.9%;
Liu et al.	2025	526	CT + Single-center retrospective + pathology	Radiomics + clinical + pathology	LASSO + logistic regression + SVM	Predicts recurrence pattern	AUC 0.83–0.825 Sensibility 91.1–94.1% Specificity 68.8–80.8%
Lin et al.	2026	239	Clinical	Clinical	XGBoost	Predicts complications	AUC 0.863 Sensibility 80.0% Accuracy 82.0%
Klontzas et al.	2024	471	CT + clinical	Radiomics + clinical	XGBoost	Predicts complications	AUC 0.792 Sensibility 65.22% Specificity 77.46%
Bronsert et al.	2020	922	Single-center NSQIP + EHR	Clinical	Elastic-net	Predicts complications	AUC 0.93 Sensibility 83% Specificity 88% Accuracy 88%
Yunsong Liu et al.	2025	485	CT + clinical	Radiomics + clinical	DELRN	Predicts recurrence and risk	AUC 0.943 Sensibility 84.5% Specificity 93.5% Accuracy 77.6–81.1%
Zihan Zhao et al.	2025	344	CT, HE, clinical	Radiomics + clinical + pathology	eSPARK	Predicts pCR	AUC 0.803
Sebastian Djerf et al.	2026	1846	NREV, 2005–2018	Clinical	XGBoost + SHAP(XAI)	Predicts mortality rate Predicts anastomotic leakage	AUC 0.95
Jia Fu et al.	2025	741	CT	Radiomics	Vision-Mamba	Predicts pCR	AUC 0.83–0.92 Accuracy 83–91%
Junxi Hu et al.	2025	1,549	Clinical	Clinical	GBM + Boruta + LASSO + ADASYN	Predicts anastomotic leakage	AUC 0.872 Sensibility 85.4% Specificity 79.2% Accuracy 80.8%
Yuxin Zhang et al.	2024	202	CT	Radiomics	LASSO + RFE + RF	Predicts anastomotic leakage	AUC 0.89 Sensibility 43–64% Specificity 93–99% Accuracy 85–92%
Jorn-Jan van de Beld et al.	2024	417	Clinical	Clinical	LSTM + logistic regression + ResNet18	Predicts anastomotic leakage and pneumonia	Predicts anastomotic leakage: AUC 0.93 predicts pneumonia: AUC 0.85
Zhong-Wen Sun et al.	2020	205	Questionnaire survey		logistic regression	Predicts anastomotic leakage	AUC 0.946 Sensibility 83.3% Specificity 91.2%
van Kooten et al.	2022	845	DUCA	Clinical	logistic regression + LASSO + SVM + KNN + RF + AdaBoost	Predicts anastomotic leakage Predicts lung complication	Predicts anastomotic leakage: AUC 0.619 Predicts lung recurrence: AUC 0.644
Li et al.	2024	213	CT + Clinical	Radiomics + clinical	Delta radiomics + LASSO + logistic regression + XGBoost	Predicts anastomotic leakage	AUC 0.856
Xu et al.	2025	310	Clinical	Clinical	SVM + TNM	Predicts recurrence pattern	AUC 0.847 Sensibility 81.12% Specificity 71.86% Accuracy 76.13%
Liu et al.	2026	862	Clinical	Clinical	CatBoost + SHAP	Predicts lung complication	AUC 0.888 Sensibility 79.1% Specificity 84.6% Accuracy 80.8%
Lin et al.	2025	239	Clinical	Clinical	XGBoost	Prediction of Clavien-Dindo grade ≥II complications within 90 days after neoadjuvant therapy followed by esophagectomy	AUC 0.86 Sensibility 80% Accuracy 82%
Yang et al.	2025	356	Clinical	Clinical	LightGBM	Predicts anastomotic leakage	AUC 0.956 Sensibility 90.05 Specificity 85.5%
Deng et al.	2026	443	CT + clinical	Radiomics + clinical	GBDT+RSF + Cox regression	Predicts survival	AUC 0.854
Parikh et al.	2025	4,228	Medical record	Clinical	Cox regression + logistic regression + RF + XGBoost	Predicts complication	AUC 0.79–0.96
Ubels et al.	2023	1,509	Clinical	Clinical	logistic Regression	Predicts anastomotic leakage	AUC 0.79
Klontzas et al.	2024	471	CT + clinical	Radiomics + clinical	XGBoost	Predicts anastomotic leakage	AUC 0.792 Sensibility 65.22% Specificity 77.46%
van Nieuw Amerongen et al.	2024	108,208	Clinical	Clinical	Cox regression + logistic regression	Predicts recurrence pattern	AUC 0.6–0.79
Takeuchi et al.	2025	130	RAMIE video of surgery	Video	CNN	Predicts nerve injury	Sensibility 84.4%
Zhang et al.	2025	741	CT + clinical	Radiomics + clinical	Vision-Mamba	Predicts pCR	AUC 0.83–0.86 Sensibility 73–82% Specificity 84–100% Accuracy 83–91%

## 3 Results

### 3.1 AI-driven postoperative complication prediction and early intervention

Traditional postoperative monitoring for ESCC often depends on subjective symptom reporting and scheduled imaging, which can lead to delays in addressing complications and an inefficient use of healthcare resources. Leveraging artificial intelligence (AI), researchers have developed multi-modal predictive models that integrate electronic health records (EHRs), radiomics, and biomarkers to achieve high precision in forecasting postoperative complications.

Several studies demonstrate the potential of AI in identifying hidden risk factors and early preclinical signals of complications such as anastomotic leakage (AL), pneumonia, and other adverse events. An artificial neural network (ANN)-based algorithm, for example, was able to analyze 27 clinical variables, including preoperative hemoglobin levels and surgical duration, achieving an early complication prediction accuracy of 89.3%, with an average lead time of 3–5 days compared to conventional methods (Dichter et al., 2024; Oyama et al., 2020). Furthermore, the integration of dynamic changes in FDG-PET-CT standardized uptake values (SUV) with AI algorithms has shown promise in optimizing treatment decisions for patients undergoing arterial infusion chemotherapy (AIC), as SUV changes were strongly correlated with improved local control rates (*p* < 0.05) (Li et al., 2025). This field has yielded excellent results in postoperative supervision for lung cancer and oral cancer, among others. Additionally, further applications in esophageal cancer show significant potential.

For predicting recurrence risk after surgery, the most common approach involves combining CT/PET-CT radiomics with machine learning classification or survival models. One study developed a logistic regression and support vector machine (SVM)-based model integrating preoperative CT radiomic features with clinical variables. This model demonstrated robust performance in predicting 3-year recurrence probabilities (AUC: 0.826 in the training cohort, 0.830 in the validation cohort) and recurrence patterns (e.g., local, regional, or distant) across 526 patients (Liu et al., 2025). By refining risk stratification within the same TNM stage, such models address the issue of “stage heterogeneity” and provide tailored recommendations for surveillance intensity and adjuvant therapies.

In addition to imaging-focused approaches, multi-modal frameworks incorporating clinical and pathological variables, nutritional/inflammatory markers, response to neoadjuvant therapy, and postoperative complication trajectories have emerged. Explainable AI models (e.g., SHAP analysis) have highlighted key predictors and reduced the “black-box” limitations of traditional machine learning systems (Lin et al., 2026). For instance, one study identified prognostic nutritional index (PNI), carcinoembryonic antigen (CEA), age, and lymphocyte-to-monocyte ratio (LMR) as critical variables, achieving an AUC of 0.86 in predicting postoperative complications (Lin et al., 2026). Such models help clinicians implement targeted risk reduction strategies, such as enhanced drainage, respiratory rehabilitation, or intensified follow-up. Another emerging trend is the use of AI-guided endoscopic response assessment in organ-sparing protocols, particularly for detecting residual disease or subtle signs of recurrence. For example, in patients undergoing active surveillance after non-radical resection, AI-driven endoscopic assessments have shown potential to improve sensitivity in detecting early-stage recurrence and guiding salvage therapy (Bondzi-Simpson et al., 2026; van der Wilk et al., 2025).

### 3.2 Clinical value of intelligent follow-up systems

AI-powered disease management platforms that integrate natural language processing (NLP) and machine learning are transforming traditional follow-up pathways. Such platforms often include three core functionalities: (1) automated symptom monitoring (e.g., via digitized questionnaires like the Leicester Cough Questionnaire), (2) stratified alert systems (e.g., automated trigger alerts based on classification scores), and (3) personalized health education modules (e.g., customized rehabilitation plans) (Xie et al., 2021).

Clinical studies suggest that AI platforms improve the efficiency of postoperative monitoring by proactively identifying issues. For example, AI-enabled systems enhanced the recognition of postoperative issues within 7 days by 40%, with patients reporting improved satisfaction and communication quality with healthcare providers (Xie et al., 2021). Advanced navigation and three-dimensional visualization technologies integrated into AI systems have also improved surgical precision, reducing blood loss and postoperative scarring, and enhancing patient outcomes (Yan et al., 2024).

For complications such as anastomotic leakage and pulmonary complications, multi-modal machine learning models that integrate clinical data, laboratory results, and imaging findings have demonstrated high accuracy in early warning detection. For instance, a recent study employed XGBoost to predict AL with an AUC of 0.79, 77.46% specificity, and 65.22% sensitivity. The use of explainable AI (SHAP analysis) helped identify key variables, including PNI, age, and CEA levels, providing actionable insights for stratified interventions (Klontzas et al., 2024). These models allow for dynamic surveillance, where high-risk patients can be closely monitored with additional imaging, laboratory tests, or clinical evaluations.

For real-world follow-up scenarios, NLP tools have proven effective in identifying complications from unstructured clinical texts, such as discharge summaries and follow-up notes. A retrospective study on postoperative NSQIP data of 6,840 patients achieved an AUC of 0.93 for identifying complications, using elastic-net regression and ICD/CPT codes as features (Bronsert et al., 2020). The ability to mine existing clinical notes without additional data collection costs is a significant advantage, and when combined with structured data, these models offer improved sensitivity and timeliness.

### 3.3 Multimodal data integration and decision optimization

The advent of third-generation AI models, including generative AI architectures like GPT-4, holds promise for addressing the complex challenges of ESCC postoperative care. These models can integrate pathology reports, imaging data, and patient-reported outcomes (PROs) to generate comprehensive, actionable insights for clinical decision-making (Teo et al., 2025; Howell et al., 2024). For instance, AI models have been used to optimize anesthesia strategies by analyzing different regimens’ molecular impacts on vascular endothelial growth factors, offering a personalized approach to perioperative management (Yan et al., 2018). In virtual emergency scenarios, large foundational models have achieved diagnostic accuracy comparable to or exceeding primary care physicians, with a high-quality recommendation rate of 77.1% compared to 67.1% for general practitioners (Zeltzer et al., 2025).

Wearable technologies have also been explored for remote postoperative monitoring. A feasibility study using wearable patch sensors for home-based vital sign monitoring after esophagectomy demonstrated that daily monitoring improved clinician-patient communication without increasing patient burden. Although vital sign trends did not alter clinical decisions within this study, patients appreciated the added layer of care and monitoring (Breteler et al., 2020). Additionally, wearable fitness trackers (WFTs) have shown promise for managing postoperative recovery and activity levels, with evidence of reduced pneumonia rates, shorter hospital stays, and improved nutritional outcomes among patients using WFTs compared to traditional recovery pathways (Honke et al., 2022).

Patient-reported outcomes (ePROs) have emerged as a critical component of AI-based postoperative care. Mobile health applications that collect structured symptom data and enable real-time intervention have been well-received by patients, with high usability and satisfaction rates. For example, in a study assessing the feasibility of an ePRO tool for esophagectomy patients, the system successfully identified issues early and provided a higher level of perceived care quality (Chlan et al., 2022). These tools offer high-frequency data for time-series modeling, supporting integrated analyses of symptoms, nutrition, inflammation, and recurrence risk. By focusing follow-up resources on high-risk patients identified by predictive models, ePRO systems have the potential to optimize long-term care.

### 3.4 Advanced AI-based postoperative risk prediction and multimodal surveillance

Postoperative complications, particularly pulmonary events, remain a leading cause of morbidity after esophagectomy, contributing to prolonged hospitalization, reintubation, and increased mortality. Traditional risk scores, such as the Ferguson pulmonary risk score, utilize variables including age, performance status, FEV1%, and DLCO% to stratify patients into risk categories. External validation demonstrated moderate discriminative ability, with an AUC of 0.762 for predicting major pulmonary events and strong concordance between predicted and observed outcomes, confirming its utility for preoperative risk stratification and perioperative planning (Reinersman et al., 2016).

Recent advances leverage explainable machine learning (ML) models to enhance early prediction of postoperative pulmonary complications (PPCs). Liu et al. developed a CatBoost-based model integrating preoperative, intraoperative, and postoperative features, validated on both internal and external cohorts. The model achieved an AUC of 0.888 in external validation, demonstrating robust predictive performance. SHapley Additive exPlanations (SHAP) enabled transparent interpretation of feature contributions, identifying surgical duration, neoadjuvant therapy, and advanced age as the most influential predictors (Liu and Wang, 2026). This framework provides actionable insights for individualized perioperative management, including targeted respiratory care and early intervention for high-risk patients.

In minimally invasive esophagectomy (MIE), procedure-related morbidity remains substantial (30–40%), with pulmonary, cardiac, and anastomotic complications predominating (Parikh and Kumar, 2025). Predictive nomograms combine patient, tumor, and surgical factors to estimate individualized risk and guide perioperative decision-making. Key predictors include: (1) patient-related factors such as age >65 years, male sex, extreme BMI, ASA/ECOG score >1, malnutrition, and pre-existing comorbidities (COPD, diabetes, cardiac disease); (2) disease-related factors including tumor size >4 cm, upper thoracic location, advanced stage, and neoadjuvant chemoradiotherapy; and (3) surgical factors such as open versus minimally invasive approach, patient positioning, surgical duration, extent of lymphadenectomy, anastomotic technique, and conduit selection (Parikh and Kumar, 2025). Recent AI- and ML-enhanced models, including CatBoost, XGBoost, and neural networks, improve predictive accuracy over traditional nomograms, with AUCs ranging from 0.72 to 0.96 for outcomes including pulmonary complications, anastomotic leaks, and overall morbidity (Parikh and Kumar, 2025).

Augmenting predictive models, AI-driven platforms increasingly integrate patient-reported outcomes (ePROs) and wearable devices to capture real-time physiological and symptom data. Wearable fitness trackers and patch sensors monitor activity, vital signs, and early indicators of complications, improving clinician–patient communication and adherence to recovery protocols without increasing patient burden (Parikh and Kumar, 2025; Deboever et al., 2024; Abou Chaar et al., 2024). High-frequency longitudinal ePRO data support time-series modeling, enabling dynamic adjustment of follow-up intensity based on individualized risk trajectories.

The integration of AI, ML, and nomograms facilitates personalized surveillance, proactive intervention, and data-driven perioperative decision-making. By focusing monitoring resources on high-risk patients, these approaches can reduce unnecessary investigations for low-risk individuals, optimize follow-up schedules, and improve outcomes (Parikh and Kumar, 2025; Deboever et al., 2024; Abou Chaar et al., 2024).

### 3.5 Advanced predictive models and integration of multimodal data for postoperative management

Recent studies such as those conducted by Li et al. (2024) and Klontzas et al. (2024) have expanded the scope of postoperative surveillance in ESCC by integrating multimodal data—comprising imaging, clinical, laboratory, and perioperative parameters—into advanced predictive models. These models leverage machine learning (ML) and radiomics to identify patients at high risk of postoperative complications, including anastomotic leakage (AL), pulmonary events, and mortality, thereby enabling early intervention and personalized management.

Delta-CT radiomics has emerged as a powerful approach for AL prediction. Li et al. developed a nomogram combining delta radiomics scores, post-thoracotomy pulmonary infection (PTPI), and lifestyle factors such as dietary habits (e.g., consumption of spicy hot pot), achieving high predictive efficacy (AUC 0.90) and demonstrating clinical net benefit in perioperative decision-making. Similarly, Klontzas et al. (2024) integrated preoperative CT-derived vascular features with clinical variables using an XGBoost model, achieving an AUC of 0.79 for AL prediction and highlighting the impact of ischemia-related imaging biomarkers on postoperative outcomes. These multimodal approaches allow subtle anatomical and physiological risk factors—such as aortic calcification, vessel stenosis, and perioperative inflammation—to be quantified and incorporated into individualized risk profiles (Pérez Quintero et al., 2024).

Interpretable machine learning frameworks further enhance clinical utility. Yang et al. (2025) applied a LightGBM-based model combined with SHAP analysis to predict AL, incorporating lesion length, McKeown surgery, gastrointestinal decompression (GID) volume, and prealbumin difference as key features. The model achieved excellent discriminative performance (AUC 0.956) and offered patient-level explanations to guide postoperative management. Such explainable models facilitate clinician trust and support targeted interventions for high-risk patients, including enhanced nutritional support, early imaging, or prompt drainage procedures.

Beyond complication prediction, integration of multimodal models has extended to postoperative mortality assessment. Ubels et al. (2023) developed a logistic regression model based on the TENTACLE–Esophagus study, combining patient-related factors (comorbidities, ASA/ECOG scores) with leak-specific predictors (organ failure, intrathoracic fluid collections, defect circumference), achieving a c-index of 0.79 for 90-day mortality post-AL. This model was deployed in an online platform to aid individualized clinical decision-making and risk stratification, highlighting the potential for real-time application in postoperative care.

Prospective studies, such as the PROFUGO trial, aim to operationalize these predictive frameworks in multicenter cohorts. By systematically collecting daily postoperative clinical and laboratory parameters (e.g., inflammatory markers) and applying AI-driven predictive algorithms, the PROFUGO protocol seeks to enable early detection of AL and major complications, optimizing intervention timing and improving patient outcomes (Pérez Quintero et al., 2024).

### 3.6 Real-time AI-guided intraoperative risk mitigation

Beyond predictive postoperative models, AI is increasingly applied intraoperatively to reduce surgical complications and improve patient outcomes. Explainable AI (XAI) models have demonstrated substantial advances in predicting critical endpoints such as 90-day mortality and anastomotic leakage (AL) after esophagectomy. In a Swedish national registry cohort of 1,846 patients, XAI using XGBoost outperformed traditional logistic regression, achieving an AUC of 0.95 for 90-day mortality and 0.84 for AL, compared with 0.88 and 0.74 for logistic regression, respectively (Djerf et al., 2026). SHAP analysis provided individualized risk contributions, highlighting age, perioperative bleeding, BMI, ASA grade, surgery duration, and occurrence of AL as key nonlinear predictors. Notably, BMI and age exhibited complex associations with AL risk, increasing up to certain thresholds before plateauing or declining, underscoring the importance of capturing nonlinear relationships in perioperative risk modeling (Djerf et al., 2026).

Complementing these predictive frameworks, AI is now deployed in real-time intraoperative monitoring to prevent nerve injuries that can lead to long-term morbidity. Furube et al. (2025) developed an AI system for robotic-assisted minimally invasive esophagectomy (RAMIE) capable of detecting excessive traction (ET) on the recurrent laryngeal nerve (RLN), a major cause of postoperative vocal cord palsy. Using annotated video frames from 130 RAMIE cases, the system quantified an “excessive traction risk” (ETR) in real time and provided visual alerts before any decrease in nerve integrity monitor (NIM) amplitude occurred, effectively anticipating potential nerve injury. In a validation set of 10 surgeries, the AI correctly identified 84.4% of ET events and displayed ETR values proportional to traction intensity, demonstrating its capacity for proactive risk mitigation and immediate intraoperative feedback (Furube et al., 2025).

### 3.7 Advanced AI approaches for predicting postoperative recurrence, survival, and therapeutic response in ESCC

Voxel-level radiomics combined with deep learning has emerged as a particularly promising approach. Zhang et al. (2025) demonstrated that integrating voxel-level radiomic feature maps with CT imaging using a Vision-Mamba architecture enables accurate prediction of pathologic complete response (pCR) following neoadjuvant immunotherapy and chemotherapy, with training set AUC reaching 0.91 and validation set AUC 0.83–0.92. This framework preserves spatial heterogeneity and captures intratumoral microstructural patterns, facilitating individualized organ-preserving strategies such as the watch-and-wait approach for high-pCR likelihood patients (Zhang et al., 2025). Complementary work by Fu et al. (2025) also supports the use of AI-based imaging analysis for predicting response to neoadjuvant immunochemotherapy, highlighting the role of high-dimensional voxel-level features in capturing biologically meaningful heterogeneity and guiding perioperative management.

Beyond response prediction, AI models have been applied to postoperative survival and recurrence prediction. Deng et al. (2026) developed intratumoral and peritumoral radiomics-based machine learning models (GBDT and RSF), effectively stratifying patients into high- and low-risk survival groups, with an integrated AUC of 0.854 and significant survival separation (*p* < 0.001). Similarly, Xu et al. (2025) implemented a support vector machine (SVM) approach incorporating TNM staging, adjuvant therapy, tumor differentiation, and postoperative complications, achieving recurrence prediction sensitivities of up to 94% and robust stratification across test and validation cohorts.

Predictive modeling of postoperative complications has also benefited from explainable AI. Lin et al. (2026) developed an interpretable XGBoost model using preoperative nutritional, inflammatory, and laboratory markers, achieving an AUC of 0.86 for Clavien–Dindo ≥ II complications and enabling risk stratification into low-, medium-, and high-risk groups. Integration of these models with real-time monitoring and longitudinal EHR data allows dynamic surveillance, facilitating early interventions for high-risk patients and potentially reducing morbidity and readmissions (Xu et al., 2025; Lin et al., 2026).

According to a study published in Scientific Reports (2025), AI-powered models, particularly CNNs, are employed to predict the risk of ESCC through non-invasive methods, such as the analysis of soft palate endoscopic images. This research identified a technique for predicting high-risk individuals for ESCC, demonstrating the potential of utilizing small datasets to achieve high diagnostic precision, even in resource-limited environments (Waki et al., 2025). The development of the ENDOANGEL-AS system, which integrates deep learning and NLP, aims to enhance the surveillance of high-risk patients. This system can automatically identify high-risk individuals based on endoscopic and pathological reports, classify them into various risk levels, and recommend appropriate surveillance intervals. The system was evaluated against physicians and exhibited significantly improved performance in accurately assigning surveillance intervals, representing a crucial advancement in enhancing surveillance rates for upper gastrointestinal cancers and addressing issues related to inconsistency and physician workload (Li et al., 2022).

Collectively, these studies illustrate a trend toward end-to-end AI pipelines that integrate preoperative imaging, pathological data, perioperative variables, and longitudinal follow-up to inform personalized postoperative management.

### 3.8 Current limitations of the evidence base

While AI shows great promise in postoperative management for ESCC, several critical limitations must be addressed to enable its safe and effective integration into clinical care. The primary bottleneck has shifted from “whether AI can predict” to “how AI can be safely embedded into clinical workflows.” However, many studies, while demonstrating impressive performance in prediction metrics like AUROC and C-index, often fail to consider the real-world applicability of these models, particularly in clinical settings.

At first, a significant proportion of studies are retrospective and single-center, which limits the generalizability of the findings. Such studies often include limited patient populations, use different imaging protocols, and apply varied inclusion criteria, leading to high heterogeneity in results. For instance, external validation accuracy fluctuates by as much as 15–20%, reflecting inconsistencies in how AI models perform across diverse healthcare settings (Guidozzi et al., 2023; Haesevoets et al., 2025). Furthermore, the reliance on retrospective data does not always account for the dynamic and evolving nature of postoperative care, such as changes in patient management strategies or shifts in surgical techniques over time.

Then, many studies do not adhere to standardized data acquisition practices, which undermines model robustness and reproducibility. Differences in imaging protocols, laboratory testing, and even pathology processing contribute to the poor harmonization of data across institutions. The quality of data, particularly unstructured clinical text and imaging data, can also vary, affecting the model’s training and evaluation. As a result, AI systems often struggle to achieve the desired level of accuracy when applied to real-world clinical data, where noise and variability are prevalent.

A major limitation of the current literature is the lack of prospective, multicenter studies that assess AI systems in diverse, heterogeneous patient cohorts. Prospective validation is crucial to verify that models can handle the complexities and variations found in everyday clinical practice. Without such validation, the clinical translation of AI models remains speculative, and their real-world impact on outcomes such as patient survival, complication rates, and quality of life remains unclear. Furthermore, many studies do not include long-term follow-up, which is essential for evaluating the effectiveness of postoperative management and recurrence prediction over extended periods.

Another challenge that remains under-explored in the literature is the integration of AI systems into clinical workflows. Many AI models, while effective in research settings, fail to consider the real-world constraints of clinical environments, such as clinician workload, alert fatigue, and the need for interpretability. Studies that focus solely on the predictive performance of models, without addressing their practical deployment, may overlook these critical factors. For example, without prespecified clinical action thresholds and escalation protocols, AI predictions may lead to unnecessary investigations or, worse, missed opportunities for timely intervention. Moreover, the lack of real-time monitoring and updating of predictive models—especially in the context of dynamic, longitudinal data—limits the ability to provide adaptive, personalized care.

While empirical studies specifically investigating the use of third-generation AI in postoperative care for ESCC are limited, we will emphasize the broad applicability of these models across other oncological fields, demonstrating their promise in similar areas like breast cancer and lung cancer. Furthermore, we will clearly state that, although promising, the application of these advanced AI models in ESCC remains a topic of ongoing research, and their direct application to ESCC postoperative care is yet to be fully explored. We will also include a discussion about the need for future studies that could evaluate the integration of third-generation AI models into clinical practice specifically for ESCC, which will help bridge this gap.

The integration of AI in clinical care must also navigate significant ethical and privacy concerns. As AI systems become more entrenched in decision-making, ensuring the security and confidentiality of patient data will be paramount. Federated learning and other privacy-preserving approaches show promise for addressing data governance challenges, but these solutions are not yet widely implemented in clinical settings. Furthermore, AI models must be transparent and interpretable to clinicians to foster trust and ensure that AI does not replace but rather augments clinical judgment.

In summary, while the evidence base for AI in ESCC postoperative care is growing, these limitations underscore the need for more rigorous studies that evaluate not only the predictive accuracy of AI models but also their real-world applicability, safety, and impact on clinical decision-making. Future research should prioritize multicenter prospective trials, the development of standardized data protocols, and real-world testing in clinical settings. These efforts will be essential for ensuring that AI systems can be integrated into clinical care in a way that improves patient outcomes and enhances the quality of care.

## 4 Conclusion and perspectives

Artificial intelligence is rapidly reshaping perioperative care for ESCC, with the most mature evidence concentrated in postoperative surveillance and management. Across the reviewed studies, AI demonstrates consistent potential to (i) refine recurrence and survival risk stratification beyond conventional TNM staging through CT/PET-CT radiomics and machine learning, (ii) provide earlier warning of high-burden postoperative complications such as anastomotic leakage and pneumonia via multimodal models that integrate clinical, laboratory, and imaging data, and (iii) enable scalable, patient-centered follow-up through NLP-enabled EHR mining, wearable sensing, and ePRO-driven symptom tracking. Together, these components form an emerging closed-loop framework in which risk prediction is linked to continuous monitoring and timely clinical responses, offering a pathway to reduce delays, optimize resource allocation, and improve quality of life.

Looking forward, the priority is not incremental gains in AUROC but translation into executable care pathways. First, future studies should adopt harmonized endpoint definitions and reference standards for recurrence confirmation, recurrence patterns, and complication grading, enabling meaningful comparison and pooled validation. Second, multicenter prospective validation is essential, with explicit evaluation of temporal transportability, drift monitoring, and recalibration strategies as surgical techniques, imaging protocols, and systemic therapies evolve. Third, model outputs must be coupled to prespecified action thresholds and escalation rules (e.g., imaging/endoscopy triggers, nutrition and rehabilitation intensification, remote monitoring escalation), and these pathway-level interventions should be tested in embedded clinical trials using pragmatic endpoints such as readmissions, complication-related morbidity, DFS/OS, and QoL.

From a technical perspective, the next phase will likely be driven by multimodal longitudinal learning: integrating radiomics with dynamic biomarkers (e.g., ctDNA when available), laboratory trajectories, activity patterns, and symptom time series to produce continuously updated risk estimates rather than single time-point predictions. Explainable methods (e.g., SHAP) should be routinely incorporated to support auditability and clinician trust, while privacy-preserving approaches (including federated learning) may facilitate cross-institutional model development without compromising data governance. Finally, implementation science will be pivotal—addressing interoperability with hospital information systems, human factors and alert fatigue, accountability frameworks, and patient privacy concerns—so that AI-enabled follow-up can function as a safe, reliable augmentation of clinical decision-making. In sum, AI for ESCC postoperative care is transitioning from proof-of-concept to early clinical integration; with standardized evidence generation and pathway-coupled validation, it has the potential to deliver truly individualized surveillance and recovery management at scale.

## Glossary

AIC: arterial infusion chemotherapy
AI: artificial intelligence
AL: anastomotic leakage
ANN: artificial neural network
AUC: area under the curve
AUROC: area under the receiver operating characteristic curve
CAD: computer-aided diagnosis
CEA: carcinoembryonic antigen
CNN: convolutional neural network
CPT: current procedural terminology
ctDNA: circulating tumor DNA
DFS: disease-free survival
DL: deep learning
DTx: digital therapeutics
EHR: electronic health record
ePRO: electronic patient-reported outcomes
ERAS: enhanced recovery after surgery
ESCC: esophageal squamous cell carcinoma
FDG: fluorodeoxyglucose
F1-score: F1 Score (harmonic mean of precision and recall)
GPT: generative pre-trained transformer
ICD: international classification of diseases
LMR: lymphocyte-to-monocyte ratio
ML: machine learning
MoE: mixture of experts
NLP: natural language processing
NPV: negative predictive value
NSQIP: National Surgical Quality Improvement Program
OS: overall survival
PET-CT: positron emission tomography–computed tomography
PNI: prognostic nutritional index
PPV: positive predictive value
PRO: patient-reported outcomes
QoL: quality of life
RSF: random survival forest
SVM: support vector machine
SUV: standardized uptake value
TIVA: total intravenous anesthesia
TNM: tumor–node–metastasis (staging system)
VEGF: vascular endothelial growth factor
WFT: wearable fitness tracker
XGBoost: eXtreme gradient boosting
